# Viral inactivation by irradiation rays

**DOI:** 10.1038/s41377-023-01108-3

**Published:** 2023-03-14

**Authors:** Kai-Kai Liu, Chong-Xin Shan

**Affiliations:** Henan Key Laboratory of Diamond Optoelectronic Materials and Devices, School of Physics and Microelectronics, Zhengzhou University, Zhengzhou, China

**Keywords:** X-rays, Optics and photonics

## Abstract

Viral infection can lead to serious illness and death around the world, as exemplified by the spread of COVID-19. Using irradiation rays can inactive virions through ionizing and non-ionizing effect. The application of light in viral inactivation and the underlying mechanisms are reviewed by the research group of *Dayong Jin* from University of Technology Sydney.

Virus is a small, submicroscopic entity that contains only one type of nucleic acid (DNA or RNA), parasitizes live cells, and reproduces through the process of replication. Despite their tiny size, the viruses (such as human immunodeficiency virus (HIV), influenza viruses and COVID-19) can cause significant human diseases, and even lead to tens of millions death^1,2^. The battle between humans and viruses keeps ongoing for hundreds of years. Antiviral drugs can inactive virus to some extent^3,4^, but the harmful side effects and inadaptability to viral mutations severely limit their application. Alternatively, irradiation ray has been regarded as an effective tool to fight against viruses and infectious diseases caused by pathogens^5,6^. After years of endeavors, a detailed introduction on viral inactivation by irradiation rays will be effective in engineering tools for viral elimination with high efficacy, safety, and long-term application.

In the recent article published in eLight^7^, Dayong Jin from the University of Technology Sydney and Esmaeil Biazar from Islamic Azad University reviewed irradiation-rays-dependent physical methods and the corresponding underlying mechanism related to the inactivation of viruses, especially enveloped viruses (such as HIV, influenza, or hepatitis) which are responsible for serious problems in humans. The irradiation rays from gamma-ray to infrared can effect on different segments of viruses and inactivate them, as shown in Fig. 1. Irradiation rays can inactivate pathogens by destroying the genome, either directly by radiolytic cleavage of genetic material or indirectly by the action of radicals on viral nucleic acids, while causing less damage to structural components, such as the protein membrane. Thus, various vaccines can be produced by illumination of irradiation rays^8,9^. In addition, X-ray, UVC (200–280 nm) can also damage the viral genome, the reports of damage to viral proteins should come into notice^10,11^. The ultraviolet (UV) radiation includes non-ionizing radiation and ionizing radiation, and low-energy UV irradiation is considered non-ionizing radiation. As a well-known method of viral inactivation, the effectiveness of UV photons as a disinfectant is highly dependent on their incident wavelength. Compared with ionizing radiation, studies on the selective inactivation of viruses using NIR sub-picosecond laser show promising results for the disinfection of viral pathogens in blood products and open novel approach for the treatment of blood-borne viral diseases in the clinic^12^. The anti-viral effect of ultra-short pulse laser at low mean irradiance employed via impulsive stimulated Raman scattering plays a key role in viral inactivation, while high-frequency resonance vibrations provoke sufficient mechanical vibrations to break non-covalent bonds and subsequently damaging virus^13^.

**Fig. 1:**
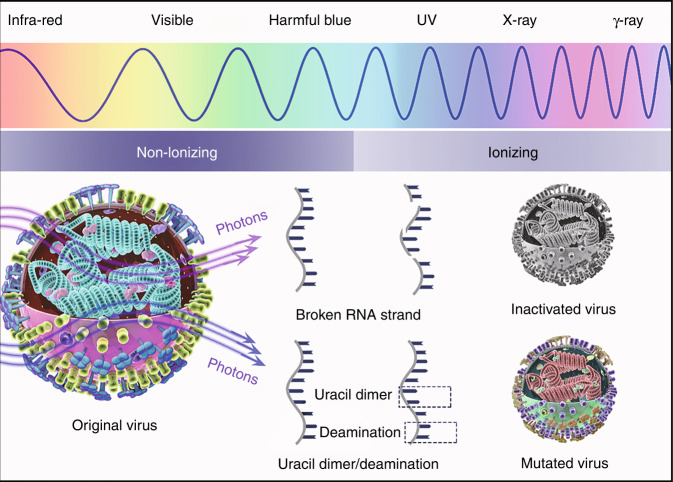
The virus inactivation and mutation by electromagnetic waves with different energies. The non-ionizing and ionizing effect caused by light with different wavelengths (top), and RNA strand broken, uracil dimmer formation and deamination during the interaction between photons and virus (bottom)

The researchers reviewed the significant advances in viral inactivation by irradiation ray (including photons, electrons, and neutrons), and discussed the corresponding underlying mechanisms and key parameters. The productive experiments and limitations on viral inactivation by irradiation ray were demonstrated. The light-induced viral inactivation mechanism and investigation will provide an effective design scheme for developing viral inactivation tools.
